# MOB2 suppresses GBM cell migration and invasion via regulation of FAK/Akt and cAMP/PKA signaling

**DOI:** 10.1038/s41419-020-2381-8

**Published:** 2020-04-14

**Authors:** Ke Jiang, Gang Yao, Lulu Hu, Yumei Yan, Jia Liu, Ji Shi, Youwei Chang, Ye Zhang, Dapeng Liang, Dachuan Shen, Guirong Zhang, Songshu Meng, Haozhe Piao

**Affiliations:** 1 Department of Neurosurgery, Cancer Hospital of China Medical University, Liaoning Cancer Hospital & Institute, Shenyang, China; 2 Institute of Cancer Stem Cell, Dalian Medical University Cancer Center, 9 Lvshun Road South, 116044 Dalian, China; 3 The First Department of Ultrasound, the First Affiliated Hospital to Dalian Medical University, No. 222 Zhongshan Road, 116021 Dalian, China; 4 Department of Oncology, Affiliated Zhongshan Hospital of Dalian University, 116004 Dalian, China; 5 Central laboratory, Cancer Hospital of China Medical University, Liaoning Cancer Hospital & Institute, Shenyang, China

**Keywords:** CNS cancer, Integrins, Cell invasion

## Abstract

Mps one binder 2 (MOB2) regulates the NDR kinase family, however, whether and how it is implicated in cancer remain unknown. Here we show that MOB2 functions as a tumor suppressor in glioblastoma (GBM). Analysis of MOB2 expression in glioma patient specimens and bioinformatic analyses of public datasets revealed that MOB2 was downregulated at both mRNA and protein levels in GBM. Ectopic MOB2 expression suppressed, while depletion of MOB2 enhanced, the malignant phenotypes of GBM cells, such as clonogenic growth, anoikis resistance, and formation of focal adhesions, migration, and invasion. Moreover, depletion of MOB2 increased, while overexpression of MOB2 decreased, GBM cell metastasis in a chick chorioallantoic membrane model. Overexpression of MOB2-mediated antitumor effects were further confirmed in mouse xenograft models. Mechanistically, MOB2 negatively regulated the FAK/Akt pathway involving integrin. Notably, MOB2 interacted with and promoted PKA signaling in a cAMP-dependent manner. Furthermore, the cAMP activator Forskolin increased, while the PKA inhibitor H89 decreased, MOB2 expression in GBM cells. Functionally, MOB2 contributed to the cAMP/PKA signaling-regulated inactivation of FAK/Akt pathway and inhibition of GBM cell migration and invasion. Collectively, these findings suggest a role of MOB2 as a tumor suppressor in GBM via regulation of FAK/Akt signaling. Additionally, we uncover MOB2 as a novel regulator in cAMP/PKA signaling. Given that small compounds targeting FAK and cAMP pathway have been tested in clinical trials, we suggest that interference with MOB2 expression and function may support a theoretical and therapeutic basis for applications of these compounds.

## Introduction

Glioblastoma (GBM), the grade IV astrocytoma according to World Health Organization classification scheme, is the most aggressive form of brain cancer with long-term survival of 10%^1^. The invasive characteristic of GBM is, at least in part, due to its high migratory potential to invade the surrounding tissue. Among the signaling pathways regulating cancer cell invasion and migration, focal adhesion kinase (FAK), a cytoplasmic protein-tyrosine kinase, is a key regulator of cell movement. Canonical FAK signaling is activated via phosphorylation upon stimulation by integrins and a broad range of growth factors and chemokines, linking to the formation and turnover of focal adhesions^2–4^. FAK regulates cell migration by activating three major signaling pathways, that is, the PI3K-Akt pathway, the RhoA subfamily of small GTPases and the Src-Cas-Crk pathway^2,3,5^. Enhanced FAK expression has been detected in brain cancer cells^6–9^, particularly in EGFRvIII (a truncated EGFR mutant lacking exons 2–7)-overexpressing GBM cells^10^. Importantly, a role for FAK in the promotion of GBM cell invasion and migration has been revealed^11–14^. FAK signaling is involved in the JAK2/STAT3 inhibitor-induced inhibition of cell motility in EGFRvIII-expressing glioblastoma cells^15^. Therefore, FAK may serve as a potential target for anti-invasive strategies in GBM. Indeed, several FAK inhibitors, such as PF562271 and VS-4718, are currently under investigation in clinical trials (http://www.clinicaltrials.gov/ct2/results?term=FAK, NCT00666926, NCT0184974). However, how FAK signaling is regulated in GBM is not fully understood.

Mps one binder (MOB) family proteins play diverse roles as regulators of members of the NDR/LATS kinase family^16,17^. Mammalian MOB2 can only interact with NDR1/2, but not with LATS1/2 kinases, to block NDR activation, thereby playing a role in cell cycle progression^18–21^. In addition, a recent study identified MOB2 as a key player in DNA damage response (DDR) via interaction with the DDR protein RAD50 and this activity of MOB2 seems to be independent of NDR signaling^22^. So far, although other members of MOB family including MOB1 and MOB3 have been implicated in cancer, the roles of MOB2 in cancer have not been thoroughly described. Analysis of expression of human MOB2 gene in cancer genomics datasets such as the Cancer Genome Atlas (TCGA) revealed a loss of heterozygosity (LOH) for MOB2 in more than 50% of the bladder, cervical and ovarian carcinomas and in at least 30% of cancer cell lines^23^, hinting that hMOB2 might represent a novel tumor suppressor. In addition, MOB2 regulated the neuritogenesis of a mouse neuroblastoma cell line Neuro2A via interaction with NDR2^24^, suggesting that MOB2 might play a role in brain cancer. However, whether and how MOB2 functions in GBM remains unknown.

Here, we report that MOB2 acts as a novel tumor suppressor in GBM. We show that the expression of MOB2 is markedly decreased in GBM patient specimens. Functionally, knockdown of MOB2 enhances the malignant phenotypes of GBM cells and promotes GBM cell invasion in a chick embryo chorioallantoic membrane (CAM) model. Mechanistically, MOB2 negatively regulates the FAK/Akt pathway via integrin. In addition, MOB2 participates in cAMP/PKA signaling-mediated inhibition of cell migration and invasion of GBM cells.

## Results

### MOB2 is downregulated in glioma patient samples

**Fig. 1:**
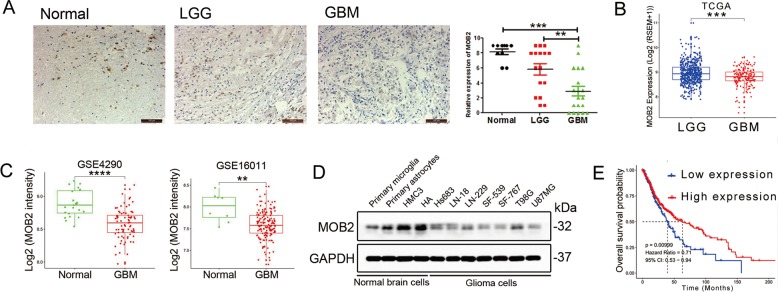
MOB2 expression is decreased in glioblastoma and associated with adverse prognosis. **a** Normal brain (*n* = 8), glioblastoma (GBM, *n* = 19) and low grade glioma (*n* = 16) samples were detected by Immunohistochemistry (IHC). Scale bar = 100 μm. Data are presented as mean ± SEM (***p < 0.001). **b** The mRNA levels of MOB2 were analyzed in glioblastoma and low grades gliomas according to the TCGA datasets. Significance level was determined using the non-parametric Mann–Whitney test. Data are presented as mean ± SEM (***p < 0.001). **c** MOB2 was analyzed in glioblastoma and normal brain samples according to GSE4290 and GSE16011 microarray datasets. Significance level was determined using the Non-parametric Mann–Whitney test. Data are presented as mean ± SEM (***p < 0.001). **d** MOB2 protein expression in normal brain and glioma cells was detected by IB. **e** Kaplan–Meier survival curves for correlation between MOB2 mRNA expression and survival of gliomas patients in the TCGA RNA-seq dataset.

To assess the expression of MOB2 in glioma clinical samples, we performed an immunohistochemical (IHC) analysis on normal brain tissues, low grade gliomas (LGGs, WHO grade I and II gliomas, *n* = 16) and glioblastoma (GBMs, WHO grade IV gliomas, *n* = 19) samples. The IHC analysis showed that MOB2 expression was largely undetected in the examined GBM samples while it was abundant in LGG samples and normal brain samples (Fig. 1a). Clinical characteristics of the patients are shown in Supplemental Table 1. Consistently, bioinformatic analyses of MOB2 mRNA expression data obtained from The Cancer Genome Atlas (TCGA) showed MOB2 mRNA levels were significantly downregulated in GBM samples (*n* = 165) compared to LGG samples (*n* = 525) in the TCGA data set (Fig. 1b; *p* = 3.94e−05, https://tcga-data.nci.nih.gov/tcga/). In addition, In addition, bioinformatic analyses of GSE4290 and GSE16011 datasets revealed that MOB2 mRNA levels ware higher in normal brain samples compared to GBM samples (Fig. 1c). Moreover, MOB2 protein expression level was lower in GBM cell lines compared to normal brain cells (Fig. 1d). To further investigate the clinical relevance of MOB2 in glioma, we performed Kaplan-Meier survival analyses of MOB2 mRNA expression data from the TCGA (*n* = 690, low expression: 173; high expression: 517). Low MOB2 expression significantly correlated with a poor prognosis for glioma patients in the TCGA data set (Fig. 1e; *p* = 0.00999).

### MOB2 overexpression suppresses, depletion of MOB2 enhances, GBM cell proliferation, migration, invasion, and clonogenic growth

**Fig. 2:**
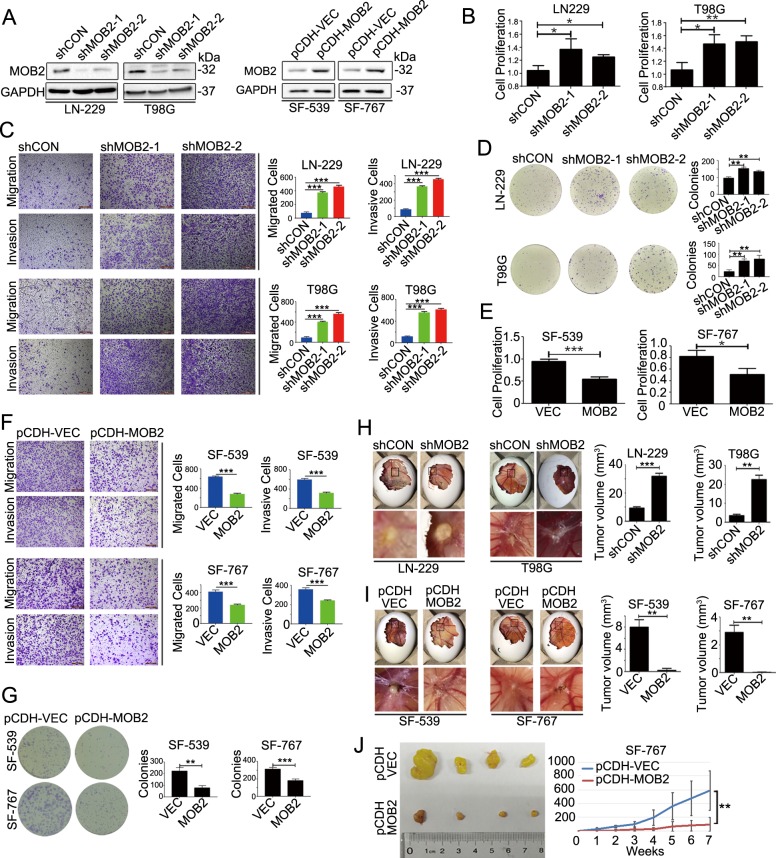
MOB2 is a tumor suppressor in GBM. **a** IB analysis of the efficiency of stable knockdown of MOB2 in LN-229 and T98G cells (shCON and shMOB2)or stable overexpression of MOB2 in SF-539 and SF-767 cells (pCDH-VEC and pCDH-MOB2), GAPDH was used as a loading control. **b** Cell proliferation rate of MOB2-depleted LN-229 and T98G cells was measured by BrdUrd labeling. **c** Representative images and quantification of migration and invasion of MOB2-depleted LN-229 and T98G cells. Scale bar = 200 μm. **d** Cell proliferation of MOB2-depleted LN-229 and T98G cells was measured by colony formation assays. **e** Cell proliferation rate of SF-539 and SF-767 cells stably expressing MOB2 was measured by BrdUrd labeling. **f** Representative images and quantification of migration and invasion of MOB2-overexpressed SF-539 and SF-767 cells. Scale bar = 200 μm. **g** Cell proliferation of SF-539 and SF-767 cells stably expressing MOB2 was measured by colony formation assays. **h**, **i** Chick embryos with CAM tumors formed from MOB2-depleted LN-229 and T98G cells (**h**) or MOB2-overexpressed SF-539 and SF-767 cells (**i**) were dissected and the size of CAM tumors was calculated. A minimum of ten embryos per condition was analyzed. **j** Enlarged tumor volumes in MOB2-overexpressed SF-767 cells vs control mice, were measured weekly (7 weeks). All experiments were performed as three independent experiments. Data are presented as mean ± SEM (*p < 0.05, **p < 0.01, ***p < 0.001).

To determine the potential function of MOB2 in GBM, we silenced MOB2 expression using two distinct shRNAs lentiviral targeting constructs in LN-229 and T98G GBM cell lines which expressed relatively high levels of MOB2 protein (designated as LN-229-shMOB2 and T98G-shMOB2 respectively; control cells infected with lentiviruses expressing scramble shRNA were designated as LN-229-shCON and T98G-shCON respectively) and stably overexpressed MOB2 with V5-tag in GBM cell lines SF-539 and SF-767 which expressed relatively low or undetectable MOB2 protein (designated as SF-539-pCDH-MOB2 and SF-767-pCDH-MOB2 respectively); control cells were designated as SF-539-pCDH-VEC and SF-767-pCDH-VEC, respectively). The effects of knockdown or overexpression of MOB2 in these cells were confirmed by immunoblot (IB) analysis (Fig. 2a). We then performed several cell-based assays to dissect the biological functions of MOB2 in GBM cells. Stable depletion of MOB2 in LN-229 and T98G cells led to a significantly potentiated capacity of the cells to proliferate (Brdu assay), migrate (Transwell migration assay), invade (Transwell invasion assay) and form colonies (colony formation assay) (Fig. 2b–d). Conversely, stable MOB2 overexpression in SF-539 and SF-767 cells resulted in opposing effects (Fig. 2e–g). In addition, the effects of MOB2 depletion on GBM cell colony formation, invasion and migration were rescued by either wild type (WT) MOB2 or the MOB2-H157A mutant which is defective in binding NDR1/2 (Suppl. Fig. 1A, B). To study MOB2-mediated effects in GBM pathogenesis in vivo, the in vivo chicken chorioallantoic membrane (CAM) model was implanted with both MOB2-depleted LN-229 and T98G cells or MOB2-overexpressing SF-539 and SF-567 cells, and their corresponding control cells respectively. In line with our in vitro findings, both LN-229-shMOB2-derived and T98G-shMOB2-derived tumors displayed enhanced invasion with tumor strands invading the chicken host tissue compared to control cell-derived tumors (Fig. 2h). In contrast, we observed decreased invasion potential in SF-539-MOB2- and SF-767-MOB2-derived tumors in the CAM model compared to control tumors (Fig. 2i). Furthermore, the microscopic features of HE (hematoxylin-eosin)-stained tumors from CAMs were basically the same: cells with a large nucleus formed a compact tumor mass (black arrows) (Suppl. Fig. 2A, B). On IHC, densely stained Ki67-positive cells (red arrows) were found in tumor sections (Suppl. Fig. 2A, B). We next extended our investigation in a xenograft model. MOB2-overexpressing SF-767 cells inoculated in nude mice showed a significant decrease in tumor growth compared to control cells (Fig. 2j). Taken together, these data suggest that MOB2 functions as a tumor suppressor in GBM.

### Depletion of MOB2 enhances the formation of focal adhesions and confers resistance to anoikis

**Fig. 3:**
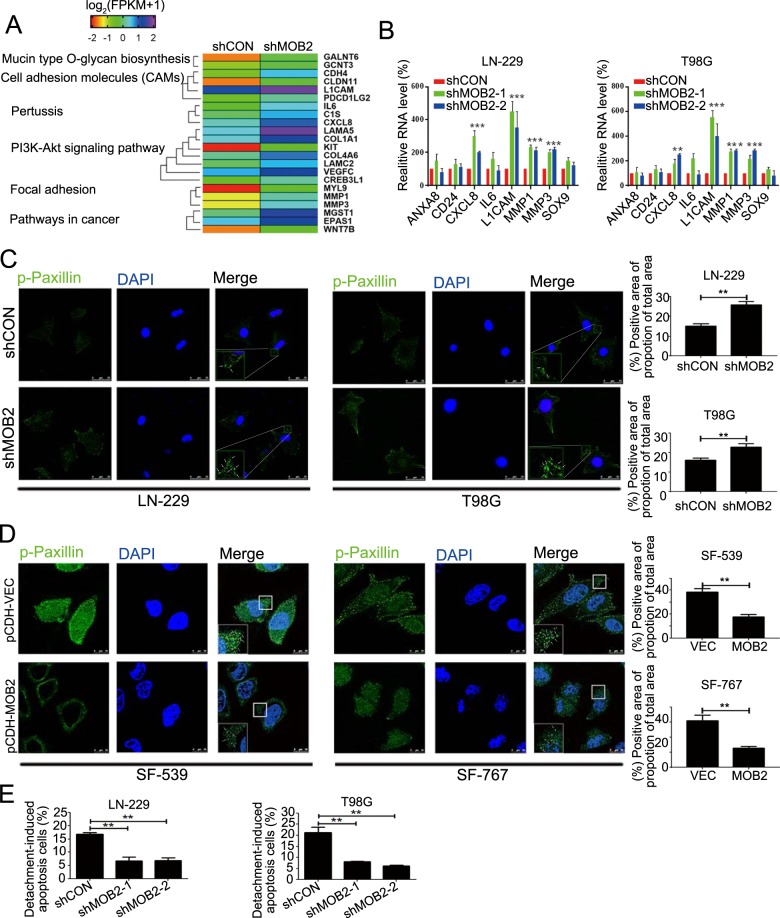
Knockdown of MOB2 promotes the formation of focal adhesions and inhibits anoikis. **a** Gene set enrichment analysis of MOB2-regulated genes and signaling pathway in LN-229 cells, raw data were submitted to NCBI (GSE139339). **b** Relative mRNA levels in LN-229-shMOB2 cells compared to the control cells (shCON). **c**, **d** LN-229 and T98G cells stably knockdowning of MOB2 (**c**) or SF-539 and SF-767 cells stably overexpressing of MOB2 (**d**) were stained with p-Paxillin (green) to indicate focal adhesions by immunofluorescence. DAPI was used for nuclear staining. The positive area of the proportion of the total area was quantified. The region enclosed in the red square has been enlarged for a clearer proportion, and the arrows indicated the proportion. Scale bars = 10 or 25 μm. **e** The capacity of migration and invasion of LN-229 and T98G cells stably knockdowning of MOB2 cells was measured by anoikis assay. All experiments in this figure were performed as three independent experiments. Data are presented as mean ± SEM (**p < 0.01, ***p < 0.001).

To gain insight into the mechanism of action of MOB2 in GBM, microarray analysis of LN-229 cells with or without MOB2 knockdown was performed. MOB2 suppression upregulated a total of 184 genes (*p*_adj_ < 0.05) and downregulated 42 genes (*p*_adj_ < 0.05) in LN-229-shMOB2 cells (Supplementary Table 2). KEGG pathway analysis revealed that the altered genes were enriched for genes related to pathways in cancer, PI3K-Akt signaling, cell adhesion molecules (CAMs), focal adhesion and cytokine-cytokine interaction (Fig. 3a). Alteration of genes encoding CXCL8, L1CAM, MMP1 and MMP3 in LN-229-shMOB2 and T98G-shMOB2 cells was further confirmed by qRT-PCR (Fig. 3b). Our array data indicated that MOB2 regulates the expression of genes related to focal adhesion-related signaling pathways, suggesting that MOB2 might affect the formation of focal adhesions in GBM cells. Indeed, stable depletion of MOB2 in LN-229 and T98G cells significantly increased a number of focal adhesions in these cells compared to their control cells respectively (Fig. 3c). On the contrary, stable overexpression of MOB2 in SF-539 and SF-767 cells led to a reduced number of focal adhesions compared to their control cells respectively (Fig. 3d). Anoikis is a type of cell death that occurs after extracellular matrix detachment and resistance to anoikis is a critical characteristic of metastatic cancer cells. Consistent with the altered genes related to the extracellular matrix by MOB2 depletion, we found that MOB2 depletion in LN-229 and T98G cells protected the cells from anoikis, leading to increased viability (Fig. 3e). In sharp contrast, SF-539-MOB2 and SF-767-MOB2 cells displayed increased anoikis (Suppl. Fig. 3A). Furthermore, treatment with the pan apoptosis inhibitor Z-VAD-FMK significantly attenuated MOB2 overexpression-induced anoikis in SF-539 and SF-767 cells (Suppl. Fig. 3B).

### Depletion of MOB2 activates FAK-Akt signaling

**Fig. 4:**
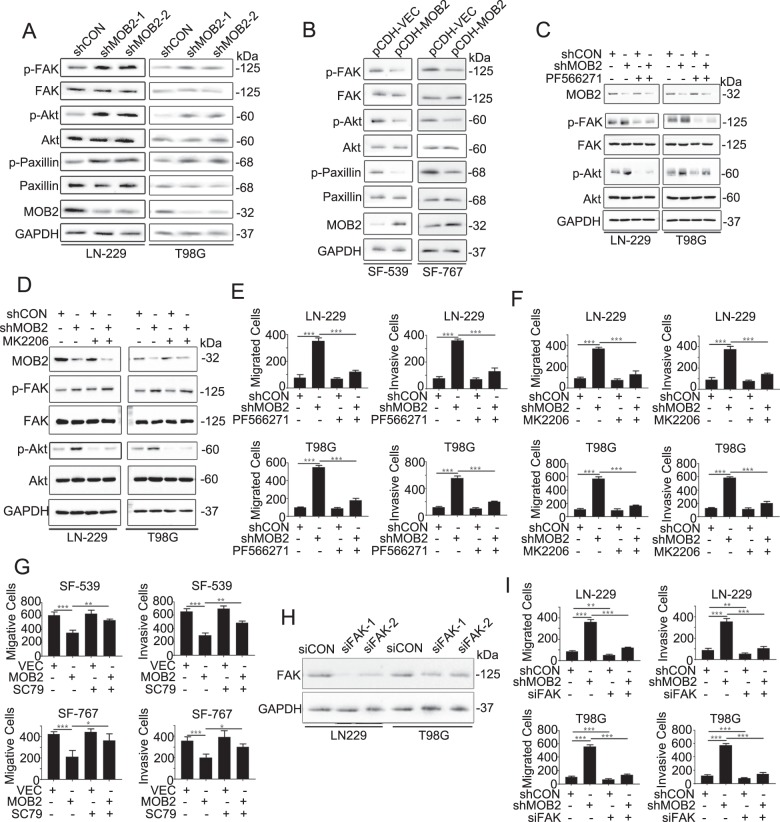
FAK-Akt signaling pathway is activated by knocking down MOB2. **a**, **b** IB with specific antibodies against p-FAK, FAK, p-Akt, Akt, p-Paxillin, Paxillin, and MOB2 in MOB2-depleted LN-229 and T98G cells (**a**), and SF-539 and SF-767 cells stably overexpressing MOB2 (**b**). GAPDH was used as a loading control. **c** LN-229 and T98G cells stably knockdowning of MOB2 or control cells were treated with vehicle (DMSO) or PF566271, IB analysis for p-FAK, FAK, p-Akt, Akt, and MOB2. GAPDH was used as a loading control. **d** LN-229 and T98G cells stably knockdowning of MOB2 or control cells were treated with vehicle (DMSO) or MK2206, IB analysis for p-FAK, FAK, p-Akt, Akt, and MOB2. GAPDH was used as a loading control. **e** LN-229 and T98G cells stably knockdowning of MOB2 or control cells were treated the same as in **c**, the ability of migration and invasion was measured by Transwell chamber assay. **f** LN-229 and T98G cells stably knockdowning of MOB2 or control cells were treated the same as in **d**, the ability of migration and invasion was measured by Transwell chamber assay. **g** SF-539 and SF-767 cells with stable overexpression of MOB2 or control cells were treated with vehicle (DMSO) or SC79, the ability of migration and invasion was measured by Transwell chamber. **h** LN-229 and T98G cells were transfected with two siFAK (siFAK-1 and siFAK-2) or siControl (siCON) oligonucleotides for 48 h, then IB analysis for FAK, GAPDH was used as a loading control. **i** LN-229 and T98G cells with a stable knockdown of MOB2 or control cells were transfected with siFAK or siControl (siCON) oligonucleotides for 48 h, then the ability of migration and invasion was measured by Transwell chamber assay. All experiments were performed as three independent experiments. Data were presented as mean ± SEM (*p < 0.05, **p < 0.01, ***p < 0.001).

FAK-Akt signaling plays a critical role in the formation of focal adhesions and cancer cell adhesion, migration and invasion. Consistent with our array data, we confirmed elevated phosphorylation levels of FAK at Y397, Akt at S473 and the focal adhesion marker Paxillin at Y118 in either LN-229-shMOB2 or T98G-shMOB2 cells by IB assay (Fig. 4a). In contrast, downregulated phosphorylation of FAK, Akt, and Paxillin was observed in SF-539-MOB2 and SF-767-MOB2 cells (Fig. 4b). Moreover, MOB2 depletion-induced activation of FAK/Akt in LN-229 and T98G cells could be reversed by the reintroduction of either MOB2 or MOB2-H157A mutant into these cells (Suppl. Fig. 4A). We next examined whether the altered FAK/Akt signaling is responsible for MOB2-regulated GBM cell migration and invasion. To this end, PF566271 (FAK inhibitor, 10 µM), MK2206 (Akt inhibitor, 2 µM) and SC79 (Akt activator, 5 µM) were employed to activate or inhibit the FAK/Akt signaling. As shown in Fig. 4c, PF566271 effectively downregulated the phosphorylation levels of both FAK at Y397 and Akt at S473 in LN-229-shMOB2 and T98G-shMOB2 cell lines, respectively. In addition, MK2206 attenuated the phosphorylation levels of Akt at S473, but it failed to decrease the phosphorylation levels of FAK at Y397 in these cells (Fig. 4d), suggesting that Akt might act downstream of FAK in these settings. In line with these inhibitory effects on FAK/Akt signaling, both PF566271 and MK2206 significantly decreased the capacity of LN-229-shMOB2 and T98G-shMOB2 cells to migrate and invade compared to mock treatments (Fig. 4e, f). In addition, overexpression of MOB2-induced decreased cell migration and invasion of SF-539-MOB2 and SF-767-MOB2 cells were profoundly reversed by treatment with SC79 (Fig. 4g). To exclude the possible off-target effects by the inhibitors, we knocked down FAK with small interfering RNAs (siRNAs) in LN-229 and T98G cells and the efficiency of knockdown was confirmed by IB assay (Fig. 4h). Knockdown of FAK achieved similar effects on cell migration and invasion to PF566271 or MK2206 (Fig. 4i).

### Integrin is critical for MOB2-regulated FAK/Akt signaling

**Fig. 5:**
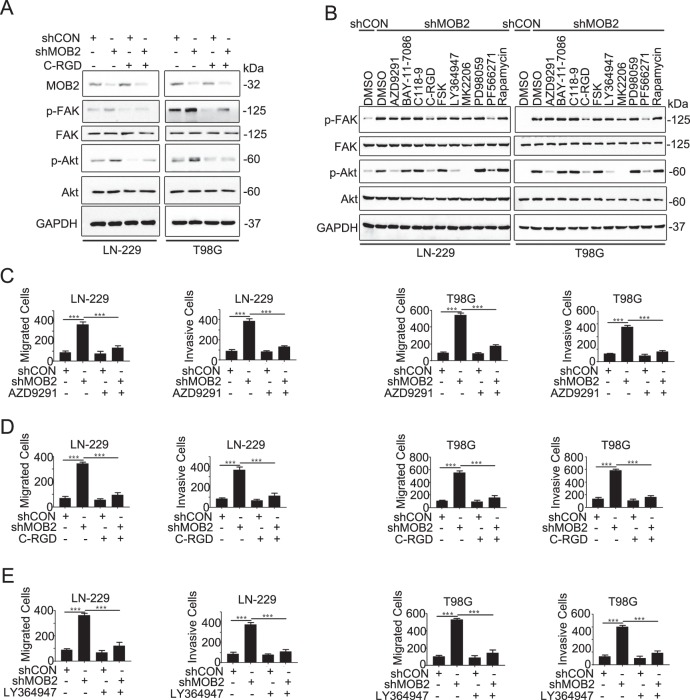
Integrin plays an important role in MOB2-regulated FAK/Akt signaling. **a** LN-229 and T98G cells with a stable knockdown of MOB2 or control cells were treated with vehicle (DMSO) or Cyclo-RGDfK (C-RGD) IB analysis for p-FAK, FAK, p-Akt, Akt, and MOB2, GAPDH was used as a loading control. **b** LN-229 and T98G cells with a stable knockdown of MOB2 were treated with vehicle (DMSO), AZD9291 (2 μM), BAY-11-7086 (10 μM), C118-9 (20 μM), C-RGD (1 μM), FSK (10 μM), LY364947 (10 μM), MK2206 (2 μM), PD98059 (10 μM), PF566271 (2 μM) or Rapamycin (2 μM), IB analysis for p-FAK, FAK, p-Akt, and Akt, GAPDH was used as a loading control. **c**–**e** LN-229 and T98G cells with a stable knockdown of MOB2 or control cells were treated with vehicle (DMSO) or AZD9291 (**c**), C-RGD (**d**) and LY364947 (**e**), the ability of migration and invasion was measured by Transwell chamber assay. All experiments were performed as three independent experiments. Data were presented as mean ± SEM (***p < 0.001).

We next investigated how MOB2 modulates FAK/Akt signaling in GBM cells. We did not find any evidence of interaction or colocalization between MOB2 and FAK or Akt in GBM cells (data not shown). As integrin is the key upstream regulator of FAK/Akt signaling, we first examined the effects of pharmacological inhibition of integrin signaling in GBM cells with MOB2 depletion. As shown in Fig. 5a, exposure to the integrin inhibitor Cyclo-RGDfK (C-RGD) led to a profound decrease in the phosphorylation levels of FAK and Akt in LN-229-shMOB2 and T98G-shMOB2 cells. We also tested whether other cell invasion-related signaling molecules/pathways would be involving in MOB2 depletion-induced activation of FAK/Akt signaling. The concentration of the inhibitors/activators used in our treatment was effective in inhibiting/promoting the activity of their corresponding target proteins without inducing significant inhibition of cell growth (data not shown). As shown in Fig. 5b, pharmacological inhibitors of EGFR (AZD9291) and TGFβ (LY364947) robustly impaired the activation of Akt but not FAK in LN-229-shMOB2 and T98G-shMOB2 cells, whereas other inhibitors such as BAY-11-7086 (NF-κB), C-188-9 (STAT3) and PD98059 (MEK1) did not display any significant effects on the activation of either FAK or Akt. These data suggest that integrin might be critical mediators for MOB2-regulated FAK/Akt signaling in GBM cells. To our surprise, Forskolin (FSK), a cAMP activator, failed to attenuate the increased activation of FAK/Akt in MOB2-depleted GBM cells (Fig. 5b). Our subsequent functional analyses indicated that C-RGD (targeting integrin), AZD9291 (targeting EGFR) and LY364947 (targeting TGFβ) significantly reversed MOB2 depletion-elevated cell migration and invasion in LN-229 and T98G cells to control cell levels (Fig. 5c–e).

### MOB2 plays a role in cAMP/PKA-mediated inhibition of cell migration and invasion

**Fig. 6:**
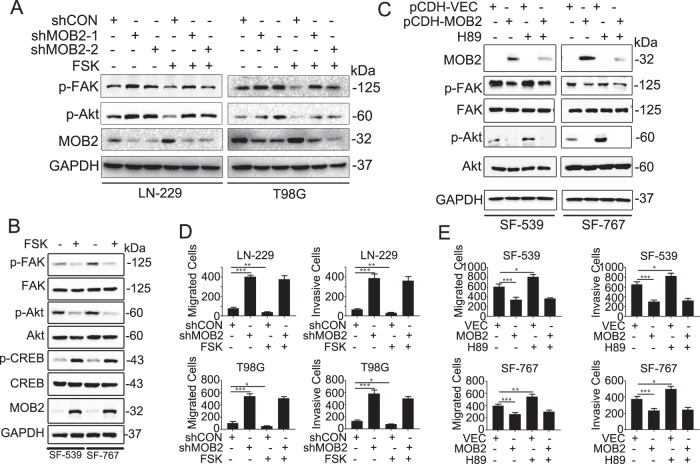
MOB2 is critical for cAMP/PKA-mediated inhibition of cell migration and invasion in glioma cells. **a** LN-229 and T98G cells with a stable knockdown of MOB2 or control cells were treated with vehicle (DMSO) or Forskolin (FSK), IB analysis for p-FAK, FAK, p-Akt, Akt and MOB2, GAPDH was used as a loading control. **b** SF-539 and SF-767 cells were treated with vehicle (DMSO) or Forskolin (FSK), IB analysis for p-FAK, FAK, p-Akt, Akt, p-CREB and CREB, GAPDH was used as a loading control. **c** SF-539 and SF-767 cells with stable overexpression of MOB2 or control cells were treated with vehicle (DMSO) or H89, IB analysis for p-FAK, FAK, p-Akt, and Akt, GAPDH was used as a loading control. **d** LN-229 and T98G cells with a stable knockdown of MOB2 or control cells were treated with vehicle (DMSO) or Forskolin (FSK), and then the ability of migration and invasion was measured by Transwell chamber assay. **e** SF-539 and SF-767 cells with stable overexpression of MOB2 cells or control cells were treated with vehicle (DMSO) or H89, and then the ability of migration and invasion was measured by Transwell chamber assay. All experiments were performed as three independent experiments. Data are presented as mean ± SEM (*p < 0.05, **p < 0.01, ***p < 0.001).

The cAMP/PKA signaling has been shown to suppress cell migration and invasion^25^, which involves the inactivation of FAK/Akt^26–30^. We further examined whether FSK affects the activation of FAK/Akt in LN-229 and T98G cells stably depleted MOB2 with two distinct shRNAs. The results indicating that MOB2 depletion impaired the inhibitory effects of cAMP/PKA signaling on the FAK/Akt pathway (Fig. 6a). We also confirmed that FSK effectively downregulated the phosphorylation levels of FAK/Akt in SF-539 and SF-767 GBM cells (Fig. 6b). As expected, FSK upregulated the Ser133 phosphorylation levels of cAMP response element binding protein (CREB) (Fig. 6b), a well characterized PKA substrate^31^, indicating a functional cAMP/PKA signaling in these GBM cells. Interestingly, FSK treatment increased the expression levels of MOB2 in both SF-539 and SF-767 cells (Fig. 6b). To further dissect the role of MOB2 in cAMP/PKA signaling, we treated GBM cells with the PKA inhibitor H89. As shown in Fig. 6c, H89 failed to upregulate the levels of p-FAK and p-Akt in SF-539-MOB2 and SF-767-MOB2 cells as in H89-treated-control parental cells. Consistently, FSK did not affect cell migration and invasion in LN-229-shMOB2 and T98G-shMOB2 cells, but significantly decreased the capacity of the corresponding control cells to migrate and invade (Fig. 6d). Moreover, exposure to H89 of SF-539-MOB2 and SF-767-MOB2 cells did not alter their capacity to migrate and invade whereas H89 treatment enhanced cell migration and invasion in control parental SF-539 and SF-767 cells (Fig. 6e).

### MOB2 interacts with PKA to enhance PKA activity

**Fig. 7:**
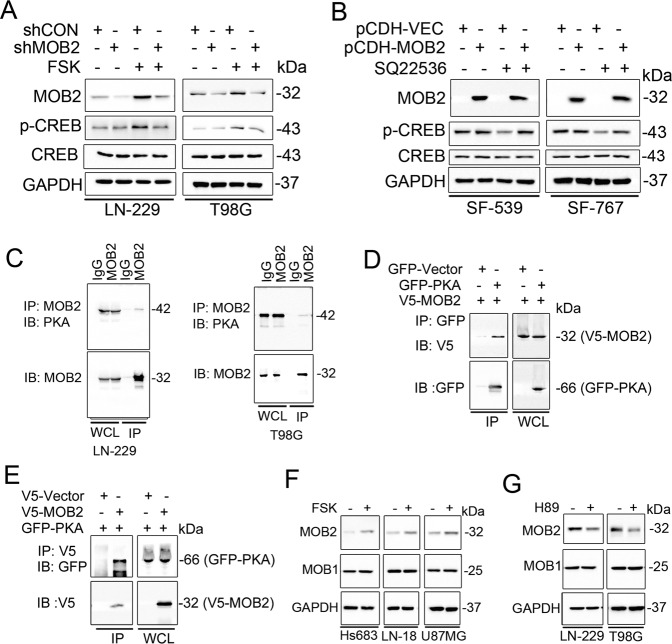
MOB2 binds to PKA and enhances PKA activity. **a** LN-229 and T98G cells with a stable knockdown of MOB2 or control cells were treated with vehicle (DMSO) or Forskolin (FSK), IB analysis for p-CREB, CREB and MOB2, GAPDH was used as a loading control. **b** SF-539 and SF-767 cells with stable overexpression of MOB2 or control cells were treated with vehicle (DMSO) or SQ22536, IB analysis for p-CREB, CREB and MOB2, GAPDH was used as a loading control. **c** LN-229 and T98G whole-cell lysates (WCL) collected from 10 cm^2^ dishes were subjected to immunoprecipitation (IP) with an anti-MOB2 antibody or an IgG control. **d**, **e** 293T cells transfected with GFP-tagged PKA (GFP-PKA) and V5-tagged MOB2 (V5-MOB2). Cell lysates were subjected to immunoprecipitation (IP) with anti-GFP (**d**) or anti-V5 antibody (**e**) and immunoblotted (IB) with the indicated antibodies. **f** Hs683, LN-18 and U87MG cells were treated with vehicle (DMSO) or Forskolin (FSK), IB analysis for MOB1 and MOB2, GAPDH was used as a loading control. **g** LN-229 and T98G cells were treated with vehicle (DMSO) or H89, IB analysis for MOB1 and MOB2, GAPDH was used as a loading control. All experiments in this figure were performed as three independent experiments.

Next, we explored the mechanisms by which MOB2 regulates the cAMP/PKA signaling in GBM cells. We hypothesized that MOB2 might affect PKA kinase activity. Figure 7a shows that treatment with FSK of LN-229-shMOB2 and T98G-shMOB2 cells failed to induce as strong phosphorylation of CREB as in control parental cells, suggesting that depletion of MOB2 impaired PKA kinase activity. In addition, treatment with SQ22536, a cAMP inhibitor, failed to downregulate the phosphorylation of CREB in SF-539-MOB2 and SF-767-MOB2 cells as in control parental cells (Fig. 7b). Of note, it appears that either overexpression or knockdown of MOB2 alone could not affect PKA kinase activity in the absence of either activation or inactivation of cAMP. Together, these data indicated that MOB2 modulates PKA kinase activity in GBM cells in a cAMP-dependent manner.

We hypothesized that MOB2 enhances PKA activity by interacting with PKA. To test this, we first determined if MOB2 could form complexes with PKA in GBM cells. Co-immunoprecipitation assay validated the endogenous interaction of MOB2 with endogenous PKA in LN-229 and T98G cells (Fig. 7c), which was also confirmed with the ectopic expression of V5-tagged MOB2 and GFP-tagged PKA at the exogenous level (Fig. 7d, e).

Our data from Fig. 6b indicated that FSK stimulation increased MOB2 levels in GBM cells, consistent with a previous study reporting that Mob2 expression in stellate astrocytes was regulated by cAMP/PKA-dependent pathway^32^. We extended and confirmed FSK-induced MOB2 expression in additional three GBM cell lines including Hs863, LN18, and U87MG (Fig. 7f). In contrast, exposure to H89 downregulated MOB2 expression in LN-229 and T98G cells (Fig. 7g). As a control, MOB1 expression was not affected by FSK or H89 in the tested GBM cells.

## Discussion

In this study, combining in vitro and in vivo analyses, we uncover MOB2 as a tumor suppressor in GBM. Bioinformatic analyses of public databases combing our IHC data indicate that the mRNA and protein expression of MOB2 are downregulated in GBM patient samples and low expression of MOB2 correlates with poor prognosis of GBM patients, suggesting that MOB2 may function as a tumor suppressor and may has a prognostic value. Furthermore, knockdown of MOB2 in GBM cells results in increased cell motility, cell proliferation, and clonogenic growth. Depletion of MOB2 in GBM cells also leads to enhanced formation of focal adhesions and resistance to anoikis. Mechanistically, MOB2 inhibits the activation of FAK/Akt pathway at least in part via integrin signaling. In addition, MOB2 is involved in cAMP/PKA signaling-regulated cell the FAK/Akt signaling and activity in GBM cells. To our knowledge, this is the first report showing the roles and mechanism of MOB2 as a tumor suppressor in GBM.

FAK is highly expressed and activated in GBM^33^, high FAK expression and phosphorylation is associated with unfavorable overall survival^8^. Given its role in GBM invasiveness^12^, FAK has been emerging as an important target for anti-invasive strategies in GBM^2,13^. However, the mechanisms for FAK regulation in GBM are not completely understood. Here we provide evidence that MOB2 negatively regulated the FAK/Akt signaling in GBM cells, which is responsible for MOB2-inhibited migration and invasion. Notably, the MOB2-H157A mutant who fails to bind NDR1/2 exhibited similar effects as its wild type counterpart, suggesting that the effects of MOB2 in GBM cells might be NDR1/2-independent. Furthermore, by using individual inhibitors targeting several cell invasion-related pathways or signaling molecules we found that the effects of MOB2 on the activation of the FAK/Akt pathway were at least in part via integrin. In addition, EGFR and TGFβ were also involved in MOB2-regulated Akt activation and activity in GBM cells. Therefore, our study indicates MOB2 as an endogenous master upstream inhibitor in the FAK/Akt pathway in GBM cells and low levels of MOB2 protein may serve as a predictive biomarker for FAK activation and inhibitor sensitivity in GBM.

A novel aspect of our study is the finding that MOB2 plays a role in cAMP/PKA signaling in GBM cells. The cAMP signaling pathway involves a number of downstream effectors, including PKA and CREB, and the recently identified EPAC-1/2^34,35^. Accumulating evidence suggests a role for the cAMP signaling in tumor biology. Early studies have established a causal role for the cAMP signaling and tumor growth inhibition in murine models^36–38^. Recent evidence shows that the cAMP signaling is commonly suppressed across many cancers including GBM^39^, suggesting a tumor suppressor role for the cAMP signaling. In GBM, recent study indicates that cAMP agonists suppressed mouse glioma growth in vivo^40^. In addition, there has been reported that some established GBM cell lines were sensitive to pharmacological activation of cAMP-induced apoptosis^39^. Importantly, cAMP pathway activation correlates with the survival of GBM patients as revealed by bioinformatic analysis of GBM patients^39^. Together, the existing evidence supports the cAMP pathway as a tumor suppressive mechanism in GBM, although the underlying mechanisms are not well understood. The data presented herein show that MOB2 regulated PKA activity in a cAMP-dependent manner. Mechanistically, MOB2 interacted with PKA. Intriguingly, cAMP levels positively regulated the expression of MOB2 in GBM cells. We thus inferred that high cAMP levels increase MOB2 expression in GBM cells, resulting in more MOB2 binding to PKA and subsequent enhanced PKA activation, whereas low cAMP levels achieve opposite effects. As the cAMP/PKA signaling is known to inhibit cell migration and invasion^26–30^, we suggest cAMP level-regulated MOB2 as a novel regulator in cAMP/PKA signaling-mediated effects on GBM cell migration and invasion.

In sum, our findings show that MOB2 suppresses GBM cell migration and invasion via regulating both FAK/Akt and cAMP/PKA pathways, thereby having some clinical implications. Several FDA-approved drugs including antidepressants, such as rolipram, have been shown to inhibit brain tumor growth in xenograft models by targeting the cAMP pathway^41^, while long-term use of tricyclic antidepressants has been associated with reduced incidence of gliomas^42^. In addition, several small compounds targeting FAK, such as PF562271 and VS-4718, have been launched for conducting clinical trials. Thus understanding the roles and mechanism of MOB2 in FAK/Akt and cAMP/PKA pathways in GBM will provide rationale and support for future therapeutic opportunities.

## Materials and methods

### Cell culture and transfection

Human GBM cell lines LN-229, T98G, LN-18, U87MG, low grade glioma Hs683, human microglia HMC3, and human embryo kidney 293T cell lines were obtained from the American Type Culture Collection, Human GBM cell lines SF-539 and SF-767 were obtained from the cell bank of the Chinese Academy of Science. Human astrocytes HA cell line was obtained from ScienCell. Mixed glial cells were isolated within 24 h from Sprague-Dawley (SD) rat pup cerebral, primary cells (10^7^ cells/75 cm^2^) were plated onto Polylysine-coated 75-cm^2^ flasks supplied with DMEM medium containing 10% FBS, adding half volume of DMEM every 2–3 days. Eight days later, supernatant (primary rat microglia) and adherent cells were collected using the shake-off method. The adherent cells were added with l-Leucine methyl ester hydrochloride for 1 h, and washed with PBS for one time, then added with DMEM medium with 10% FBS for use (primary rat astrocytes). T98G, U87MG, and HMC3 cells were routinely maintained in MEM medium with 10% FBS. LN-229 and other cells were cultured in DMEM medium with 5 and 10% FBS, respectively. All cells were maintained at 37 °C in a humidified incubator with 5% CO_2_. All cells were tested for mycoplasma contamination. Small interfering RNAs (siFAK: Two siRNA oligonucleotides were used for FAK: 5′-GCGAUUAUAUGUUAGAGAU-3′, and 5′-GUAUUGGACCUGCGAGGGA-3′) or negative control (siCON) were purchased from Genepharma (Shanghai, China) and were transfected with Lipofectamine 3000 (Invitrogen, Carlsbad, America) according to the manufacturer’s instructions.

### Antibodies and reagents

The following antibodies were purchased from Cell Signaling Technology (America): MOB1 (3863S), FAK (3285S), p-FAK (8556S), Akt (9272S), p-Akt(9271S), p-Paxillin (2541S), CREB (9197S), V5-tag (13,202S). Anti-MOB2 (PA5-75591) and Anti-V5 (66004-1) were obtained from Invitrogen Thermo Fisher Scientific (America, California). GAPDH (10494-1-AP) and GFP-tag (50430-2-AP) were bought from Proteintech (America). Paxillin (05-417) and p-CREB (06-519) were obtained from EMD Millipore (America). Ki67 was obtain from Servicebio (GB13030-2). AZD9291 (S7297), C188-9 (S8605), Cyclo-RGDfK (C-RGD, S7834), H89 (S1582), LY364947 (S2805), MK2206 (S1078), PD98059 (S1177), PF562271 (S2890), SC79 (S7863), and SQ22536 (S8283) were purchased from Selleck Chemicals (America). BAY-11-7086 was obtained from Medchemexpress (America). Forskolin (F6886) was purchased from Sigma (America). All drugs were dissolved in dimethyl sulfoxide and stored at −20 °C.

### Plasmids and lentivirus

The expression plasmids of V5 tagged-MOB2-WT (wild type) and V5 tagged-MOB2-H157A were kindly provided by Prof. Hergovich (UCL Cancer Institute, University College London, UK). GFP tagged-PKA was kindly provided by Prof. Youfei Guan (Dalian Medical University, China). To stably express MOB2 in SF-539 and SF-767 cells, V5-MOB2 was cloned into pCDH-puro lentiviral vector by PCR and the resultant plasmid was named as pCDH-MOB2. The lentiviral vectors encoding short hairpin RNAs (GIPZ-shRNAs) targeting MOB2 and scrambled shRNA were purchased from Dharmacon (America).

### Immunoblotting and immunoprecipitation

Immunoprecipitation (IP) and immunoblotting (IB) were performed as previously described^43^. For endogenous interactions, LN-229 and T98G cells grown in 10 cm^2^ dishes were harvested and the cell lysates were then subjected to IP.

### Immunofluorescence

Glioma cells were seeded on coverslips (NEST, 801008) for 24 h. Cells were fixed in 4% paraformaldehyde (PFA) and underwent permeabilization in 0.2% Triton X-100. After the blocking in 3% Bovine Serum Albumin (BSA), cells were then incubated with primary antibody for 2 h at room temperature, followed by 30 min incubation with appropriate rhodamine-conjugated secondary antibodies at room temperature. Nuclei were stained with 5 μg/mL DAPI (Sigma) in PBS. A laser scanning confocal microscope (Leica TCS SP5×) was applied to monitor the immunofluorescence (IF).

### Immunohistochemistry

Four millimeter thick paraffin-embedded tissue sections were dewaxed using a decreasing xylene/alcohol series. Briefly, the processed sections were blocked with 3% BSA and incubated with the anti-MOB2 or anti-Ki67 antibody (1:50), The DAB Detection Kit was used to develop staining signal according to the protocols provided for the streptavidin-peroxidase system (Sangon Biotech, China). Hematoxylin was used for counterstaining. All sections were investigated by light microscopy.

### Real-time PCR

RNAs were extracted with Trizol and reverse transcribed with FastKing RT Kit (Tiangen Biotech, China) according to the instructions. RT-PCR was performed using Invitrogen 2× SYBR Realtime Mix and MxPro System. Fluorescence values of each group were calculated according to ∆∆Ct. The primers of ANXA8, CD24, CXCL8, IL6, L1CAM, MMP1, MMP3, and SOX9 used for real-time PCR were supplied in Supplementary Table 3.

### Transwell migration and invasion assays

Migration and invasion experiments in this paper were carried out with a 6.5 mm diameter and 8.0 μm pore size polycarbonate membrane, which was pre-coated with (for invasion assay) or without (for migration assay) 50 μL matrigel. The cells were pre-treated mitomycin C (S8146, Selleck, America), and then the cells resuspended in 200 μL serum-free media were seeded into the upper chamber, and a total of 650 μL complete medium supplemented with 10% FBS was added into the lower chamber. After incubation at 37 °C with 5% CO_2_, the cells that passed through the membrane were fixed with 4% formaldehyde for 30 min and stained with 0.1% crystal violet for 20 min. After wiping off the upper layer of non-migrated or non-invasive cells with a cotton swab, cells were counted by light microscopy.

### Anoikis assay

Glioma cells were plated onto poly-HEMA-coated plates. The cells were collected by gentle pipetting after 72 h, and were replated on regular culture dishes for 48 h. The cells were trypsinized for manual counting.

### Colony formation assay

A suspension of glioma cells was seeded into 6-well plates and cultured in complete medium supplemented with 10% FBS for 14 days. The number of colonies (containing 50 or more cells) was counted under a light microscope.

### BrdU assay

Glioma cells were labeled with BrdU (GE Healthcare; RPN202) were performed as previously described^44^.

### Chick embryo metastasis model

Fertilized specific pathogen-free chicken eggs were obtained from Vital River Laboratory Animal Technology (Beijing, China). The eggs were incubated at 37 °C and 35% humidity. After 10 days, the eggs were randomly divided into four groups (*n* = 10) according to random numbers table, namely, shCON, shMOB2, pCDH-VEC, and pCDH-MOB2, the investigator was blinded to the group allocation during the experiment. 1 × 10^7^ glioma cells were injected to the chorioallantoic membrane (CAM) and then hatching for 8 days, images were taken and tumor sizes were measured by the following formula: *V* = 4/3 × π × *r*^3^ (*r* = 1/2 × square root of diameter 1 × diameter 2). The tumors were removed and fixed in Bouin’s solution (Solarbio, Beijing, China) for histopathological analysis with hematoxylin-eosin (HE) staining or immunohistochemistry analysis with the anti-Ki67 antibody. Chick embryos experiments were conducted at Dalian Medical University (Dalian, China), in compliance with the national guidelines for the care and use of laboratory animals. The animal study was conducted strictly following the protocol approved by the experimental animal ethics committee of Dalian Medical University.

### In vivo tumor xenograft experiment

Glioma cells (1 × 10^7^) were injected subcutaneously into flanks of female BALB/c nude mice (6 weeks old), which were maintained in animal care facilities without specific pathogens. The mice were randomly divided into two groups (*n* = 4) according to the random numbers table, namely, pCDH-VEC and pCDH-MOB2, the investigator was blinded to the group allocation during the experiment. Tumor growth was monitored using calipers where two perpendicular tumor diameters were measured weekly and tumor volume was calculated according to the formula 0.5 × length × width^2^. After seven weeks, the tumor-bearing mice were sacrificed with ether anesthesia, and xenografts were excised. Animal experiments were conducted at Dalian Medical University (Dalian, China), in compliance with the national guidelines for the care and use of laboratory animals. The animal study was conducted strictly following the protocol approved by the experimental animal ethics committee of Dalian Medical University.

### Human samples

This study was performed with approval from the Ethics Committee at the Dalian Medical University. Written informed consent was obtained from all patients and data was analyzed anonymously. Paraffin-embedded, histopathologically, and clinically diagnosed glioma samples (grade II–IV, *n* = 35) and normal tissue samples (*n* = 8) were collected at the second affiliated hospital to Dalian Medical University. The MOB2 protein expression levels in 35 paraffin-embedded glioma tissues and eight normal tissues were examined by IHC. Scoring of IHC staining was based on the intensity of staining. Scores 0–4 were used to classify the percentage of positive tumor cells (0% = 0, 1–25% = 1, 26–50% = 2, 51–75% = 3, and 76–100% = 4) and the intensity of membrane staining (negative = 0, weak = 1, medium = 2, or strong = 3). These two scores were subsequently multiplied. Low expression was defined as having final scores ≤6, and high expression was defined as final scores >6. An additional 13 frozen glioma tissues were collected from the Liaoning Cancer Hospital of China Medical University, The MOB2 protein expression levels were tested by IB, and GAPDH was used as a loading control.

### Bioinformatics analysis

RNA was extracted from shControl and shMOB2 LN-229 cells and RNA-Seq was performed by the Novogene Corporation (Beijing, China). The sequencing libraries were constructed using NEBNext® UltraTM RNA Library Prep Kit for Illumina® (NEB, USA) according to the manufacturer’s instructions. Clean data were obtained by removing reads containing adapter, reads containing ploy-N and low quality reads from raw fastq data using in-house perl scripts. Paired-end clean reads were aligned to the reference genome hg38 using Hisat2 v2.0.5. featureCounts v1.5.0-p3 was used to generate gene-level count matrix as input for edgeR’s statistical model. Differential expression analysis between shMOB2 and shControl cells was performed using the edgeR package. The *p* values were adjusted using the Benjamini & Hochberg method. Corrected *p*-value of 0.05 and absolute fold change of 2 were set as the threshold for significantly differential expression. RNA-seq data have been deposited at the NCBI Gene Expression Omnibus under the accession number GSE139339. To explore the expression pattern and prognostic implications of MOB2 in gliomas, preprocessed RNA-seq and clinical data were downloaded from UCSC XENA (TCGA-GBMLGG) (https://xenabrowser.net/datapages/). Micoarray data were obtained from Gene Expression Omnibus and ArrayExpress data repository accession number GSE4209 and E-GEOD-16011. Raw data (.cel) was processed using rma function from Bioconductor rma package with the default setting. The mas5calls function from affy package was used to generate present/marginal/absent calls for all sample replicates of all probesets. Each “present” call was assigned a value of 1.0, “marginal” was assigned a value of 0.5, and “absent” a value of 0. For averages >0.4, the probeset was considered reliable detection. Non-specific probesets that ended with “_x_at” were excluded. Filtered probesets were then mapped to the corresponding genes using hgu133plus2.db annotation package. Multiple probesets mapped to the same gene were aggregated as an average signal intensity value. Glioma patients are categorized into high and low MOB2 expression group using the 1st quartile as cutoff points (1st quartile vs. quartiles 2–4) and survival curves were based on Kaplan–Meier estimates. Differential MOB2 expression in GBM, LGG, and normal brain samples was determined by non-parametric Mann–Whitney test.

### Statistical analysis

Comparisons of data were first performed using one-way analysis of variance (ANOVA). Multiple comparisons between treatment groups and controls were evaluated using Dunnett’s least significant difference (LSD) test. For analysis of in vivo data, statistical significance between groups was calculated based on the LSD test using SPSS 17.0 software (SPSS Inc., Chicago, IL, USA). A *p*-value of *p* < 0.05 was considered statistically significant. All experiments were carried out in triplicate as three independent experiments. All statistical tests justified as appropriate and the data meet the assumptions of the tests. The variance is similar between the groups that are being statistically compared.

## Supplementary information

Supplementary Figure Legends

Supplementary Figure 1. The effects of MOB2 depletion on cell growth, cell invasion and migration were rescued by either MOB2-wild type (WT) or the MOB2-H157A mutant.

Supplementary Figure 2. Histological and immunohistological analysis in tumors from the CAM

Supplementary Figure 3. The effects of MOB2 overexpression on cell invasion and migration were treated with Z-VAD-FMK

Supplementary Figure 4. The effects of MOB2 depletion on the FAK/Akt signaling pathway were rescued by either wild type (WT) MOB2 or the MOB2-H157A mutant

Supplementary Table 1. The clinicopathological characteristics of the samples

Supplementary Table 2. Gene set enrichment analysis of MOB2-regulated genes in LN-229 cells

Supplementary Table 3. Primers used for Real time PCR
