# Supplementary material for: MOB2 suppresses GBM cell migration and invasion via regulation of FAK/Akt and cAMP/PKA signaling
Source: Cell Death Dis. 2020 Apr 14;11(4):230. doi: 10.1038/s41419-020-2381-8 (PMC7156523; doi:10.1038/s41419-020-2381-8)
Supplement: Supplementary file 1 — Supplementary Figure Legends [file 41419_2020_2381_MOESM1_ESM.docx]

**Supplementary information**

**Supplementary Figure 1. The effects of MOB2 depletion on cell growth, cell invasion and migration were rescued by either MOB2-wild type (WT) or the MOB2-H157A mutant.**

(A, B) LN-229 and T98G cells with a stable knockdown of MOB2 cells were transfected with V5-MOB2-WT (wild type) or V5-MOB2-H157A (mutation) rescue plasmid, the ability of cell growth was measured by colony formation (A), the ability of migration and invasion was measured by Transwell chamber (B). All experiments in this figure were performed as three independent experiments. Data are presented as Mean ± SEM (*p < 0.05, **p < 0.01, ***p < 0.001).

**Supplementary Figure 2. Histological and immunohistological analysis in tumors from the CAM.**

(A, B) LN-229 and T98G cells with a stable knockdown of MOB2 cells (A) or SF-539 and SF-767 cells with a stable overexpression of MOB2 cells (B) were injected to the chorioallantoic membrane (CAM) and hatching for 8 days, then the tumors were removed and fixed for histopathological analysis with H&E staining or immunohistochemistry analysis with the anti-Ki67 antibody. Scale bar=200 μm. All experiments in this figure were performed as three independent experiments.

**Supplementary Figure 3. The effects of MOB2 overexpression on cell invasion and migration were treated with Z-VAD-FMK.**

1. The ability of migration and invasion of SF-539 and SF-767 cells with a stable overexpression of MOB2 cells was measured by anoikis assay. (B) SF-539 and SF-767 cells with a stable overexpression of MOB2 cells were pre-treated with Z-VAD-FMK (Z-VAD), and then the ability of migration and invasion was measured by anoikis assay. All experiments in this figure were performed as three independent experiments. Data are presented as Mean ± SEM (*p < 0.05, **p < 0.01, ***p < 0.001).

**Supplementary Figure 4. The effects of MOB2 depletion on FAK/Akt signaling pathway were rescued by either wild type (WT) MOB2 or the MOB2-H157A mutant.**

1. LN-229 and T98G cells with a stable knockdown of MOB2 cells were transfected with V5-MOB2-WT (wild type) or V5-MOB2-H157A (mutation) rescue plasmid, IB analysis for p-FAK, FAK, p-Akt, Akt, MOB2 and GAPDH. All experiments in this figure were performed as three independent experiments.

**Supplementary table 1. The clinicopathological characteristics of the samples.**

**Supplementary table 2. Gene set enrichment analysis of MOB2-regulated genes in LN-229 cells.**

**Supplementary table 3. Primers used for Real time PCR.**
