# Supplementary figures and images for: MOB2 suppresses GBM cell migration and invasion via regulation of FAK/Akt and cAMP/PKA signaling
Source: Cell Death Dis. 2020 Apr 14;11(4):230. doi: 10.1038/s41419-020-2381-8 (PMC7156523; doi:10.1038/s41419-020-2381-8)

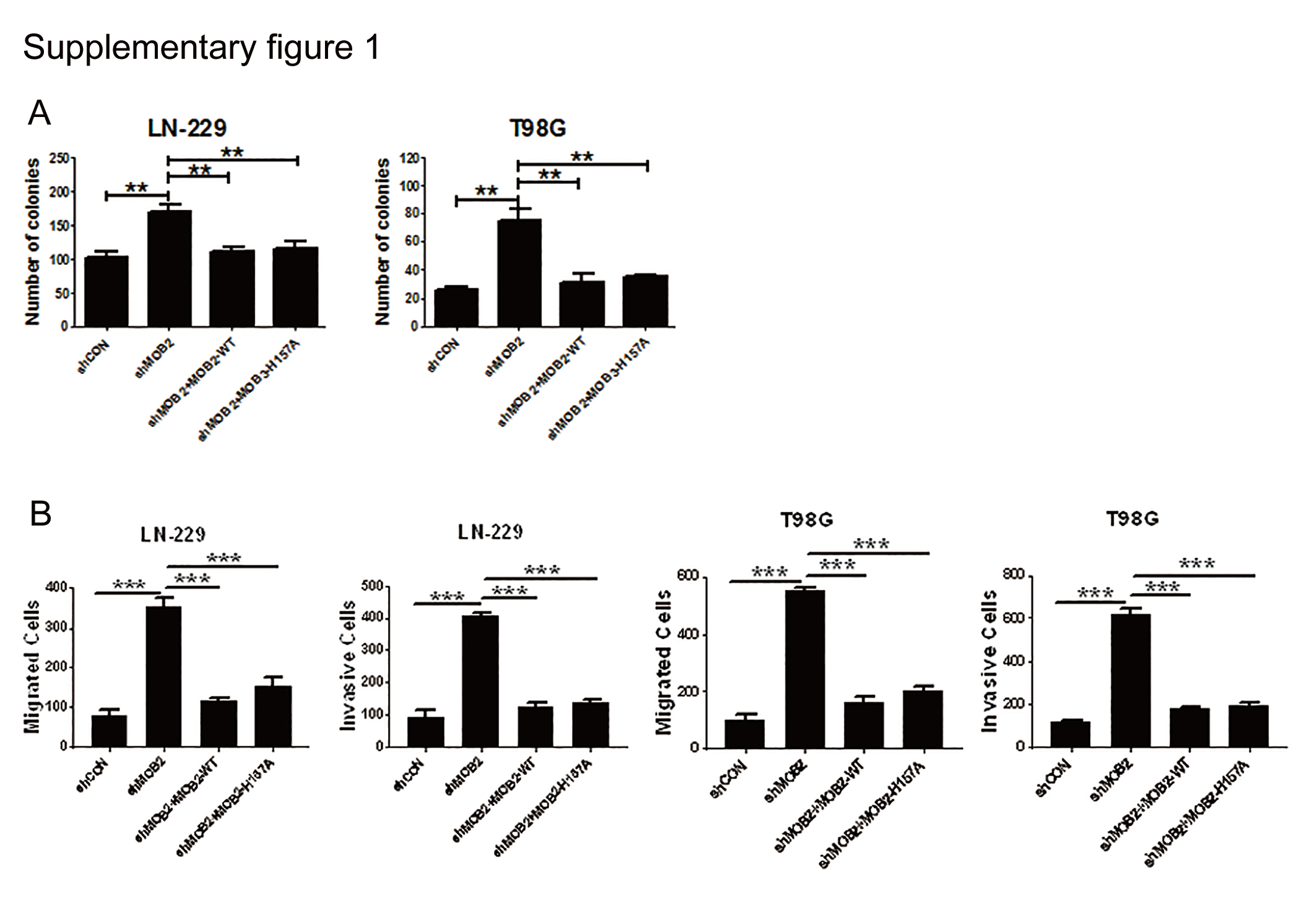

Supplement: Supplementary file 2 — Supplementary Figure 1. The effects of MOB2 depletion on cell growth, cell invasion and migration were rescued by either MOB2-wild type (WT) or the MOB2-H157A mutant. [file 41419_2020_2381_MOESM2_ESM.png]

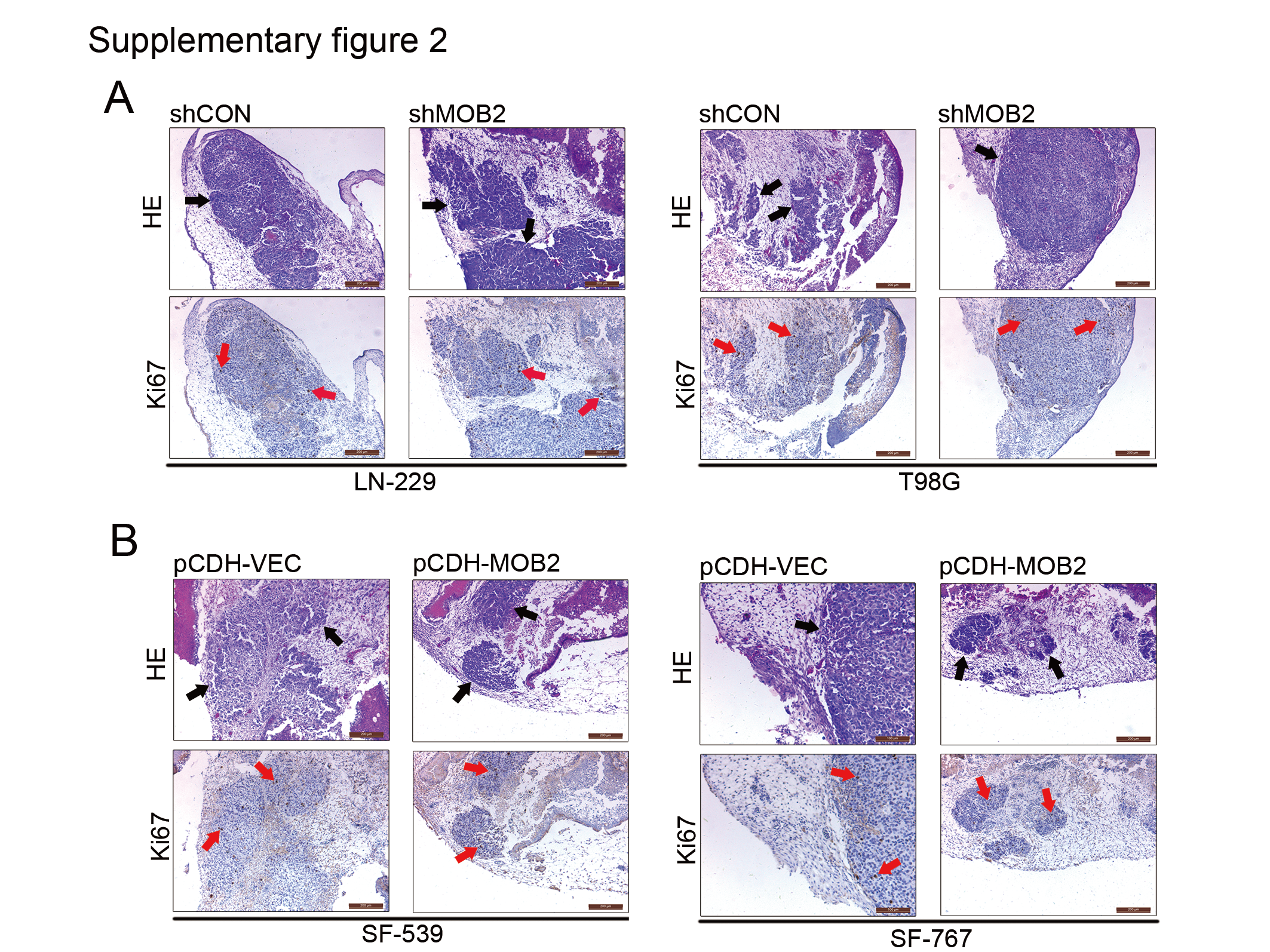

Supplement: Supplementary file 3 — Supplementary Figure 2. Histological and immunohistological analysis in tumors from the CAM [file 41419_2020_2381_MOESM3_ESM.png]

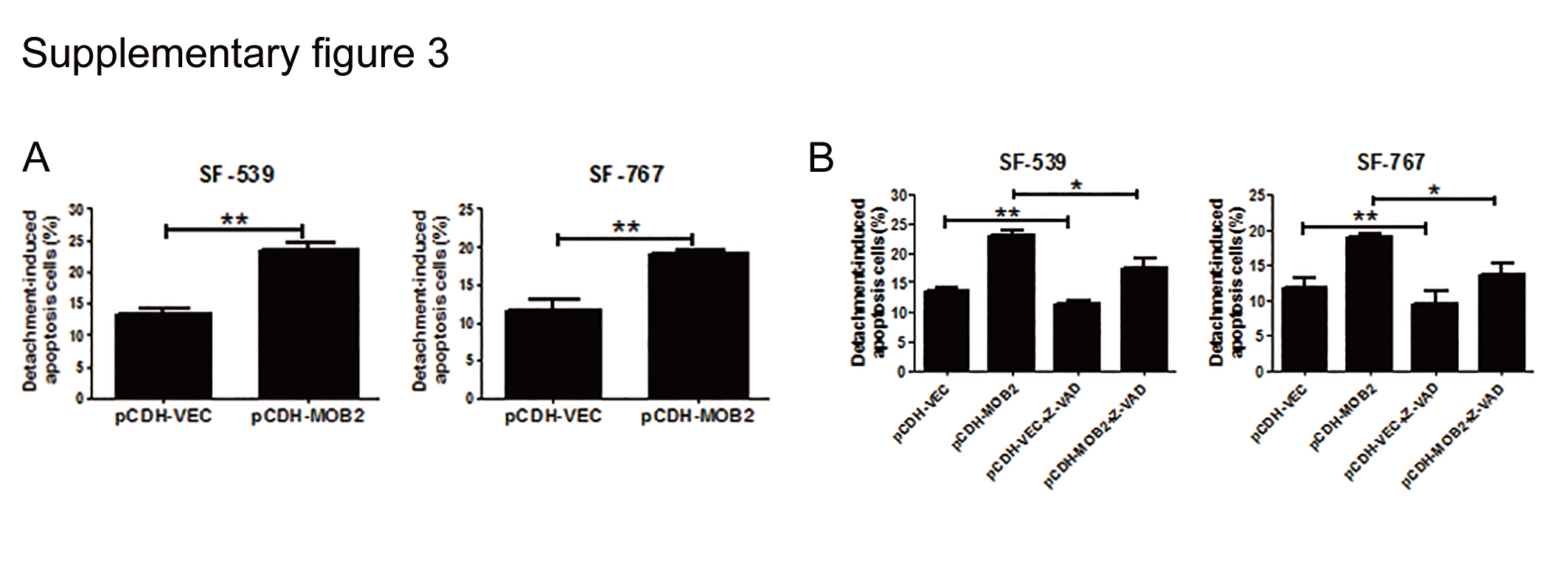

Supplement: Supplementary file 4 — Supplementary Figure 3. The effects of MOB2 overexpression on cell invasion and migration were treated with Z-VAD-FMK [file 41419_2020_2381_MOESM4_ESM.png]

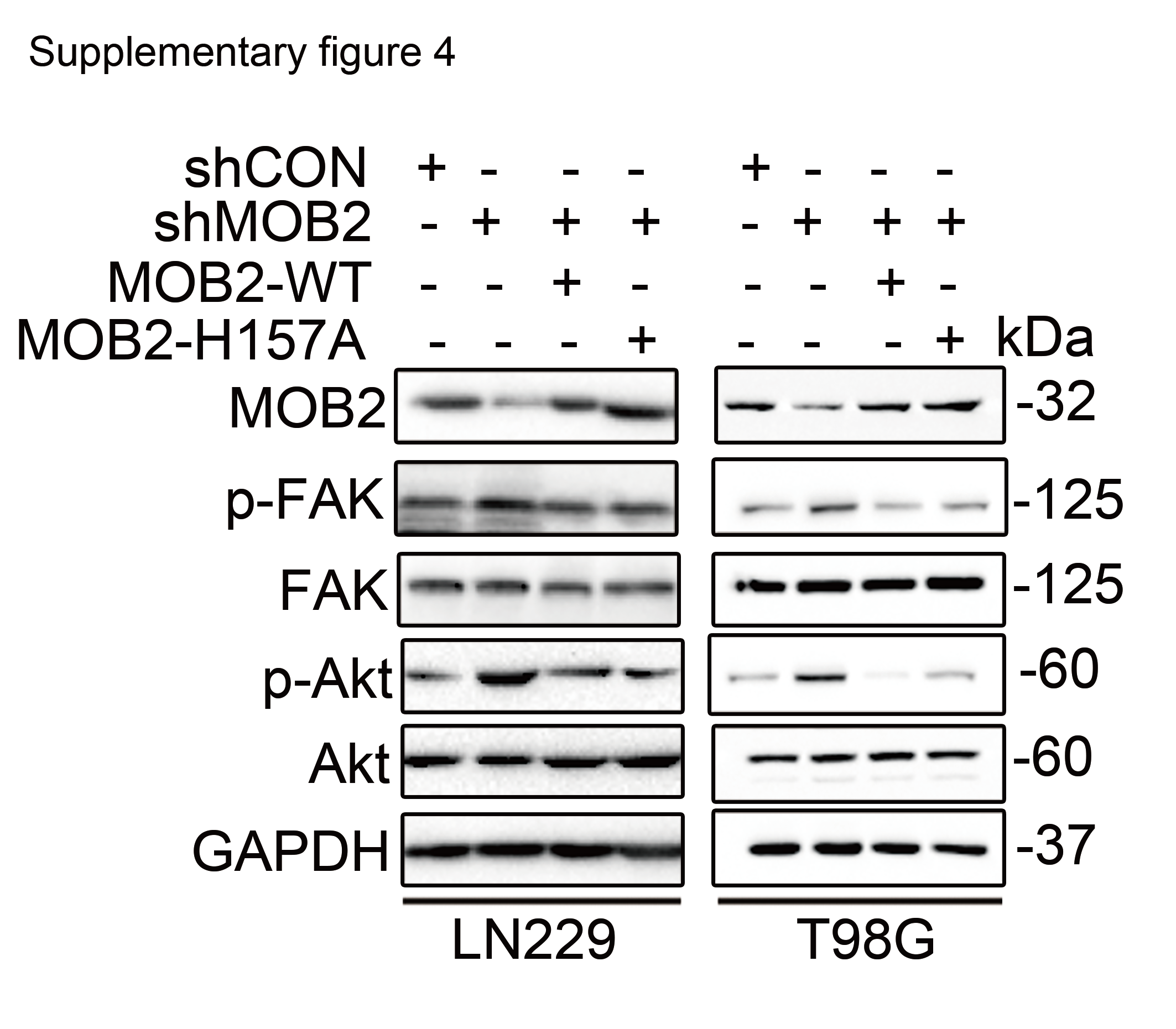

Supplement: Supplementary file 5 — Supplementary Figure 4. The effects of MOB2 depletion on the FAK/Akt signaling pathway were rescued by either wild type (WT) MOB2 or the MOB2-H157A mutant [file 41419_2020_2381_MOESM5_ESM.png]
